# Cardiac Surgery and Postoperative Atrial Fibrillation: The Role of Cancer

**DOI:** 10.3390/medicina61101815

**Published:** 2025-10-10

**Authors:** Georgios P. Georghiou, Panos Georghiou, Amalia Georgiou, Filippos Triposkiadis

**Affiliations:** 1 School of Medicine, European University Cyprus, 2404 Egkomi, Cyprus; ftriposkiadis@gmail.com; 2 Department of Cardiothoracic Surgery, Aretaeio Hospital, 2414 Nicosia, Cyprus; 3 Sackler Faculty of Medicine, Tel Aviv University, Tel Aviv 6997801, Israel; 4 Barts and The London School of Medicine and Dentistry, London E1 2AD, UK; panos.georghiou@gmail.com; 5 Department of Cardiology, Marien Hospital Düsseldorf, 40479 Düsseldorf, Germany; amalia.georgiou.med@gmail.com

**Keywords:** postoperative atrial fibrillation, cancer, cardiac surgery, arrhythmia, inflammation, cardiotoxicity, hypercoagulability, risk stratification, multidisciplinary care

## Abstract

*Background*: Postoperative atrial fibrillation (POAF) is the most frequent arrhythmic complication following cardiac surgery, affecting nearly 20–30% of patients. While conventional risk factors such as age, hypertension, and atrial enlargement are well known, emerging evidence suggests that cancer itself constitutes a significant, yet underrecognized, contributor to POAF risk. *Objective*: This review aims to systematically examine the association of cancer with POAF, explore underlying pathophysiological mechanisms, and discuss clinical implications for risk stratification and management in cardiac surgical patients with concurrent or historical malignancies. *Methods*: A comprehensive review of recent literature was conducted using PubMed and Scopus databases. Studies focusing on the epidemiology, mechanisms, and clinical management of POAF in patients with cancer were evaluated. AI-assisted tools (OpenAI’s ChatGPT) were used for formatting the graphical abstract. *Results*: Lung, breast, gastrointestinal, hematologic, and prostate cancers demonstrate the strongest association with POAF. The arrhythmogenic mechanisms include systemic inflammation, hypercoagulability, direct cardiotoxicity from cancer therapies, autonomic dysregulation, and paraneoplastic syndromes. Integration of oncologic variables into perioperative cardiovascular care is essential for precision risk assessment and outcome optimization. In a recent prospective cardiac surgery cohort, active or historical cancer independently conferred ~4-fold higher odds of POAF (adjusted OR: 3.85, 95% CI: 1.54–9.66), with cancer present in 15% of POAF cases versus 4% of non-POAF patients. *Conclusions*: Cancer represents a pivotal and multifactorial risk factor for POAF after cardiac surgery. Recognizing its role calls for a multidisciplinary approach that aligns oncologic and cardiovascular care to mitigate arrhythmic risk and improve surgical outcomes.

## 1. Introduction

As cardiovascular and oncologic diseases increasingly overlap in aging populations, the relationship between atrial fibrillation (AF) and cancer has garnered greater research attention [1,2,3]. In terms of prevalence, AF is the most observed sustained arrhythmia among cancer patients, contributing to adverse prognoses and complicating perioperative care [4,5]. Large cohort studies demonstrate that AF, when occurring in oncologic patients, is associated with increased rates of thromboembolic events, congestive heart failure, and all-cause mortality—even after adjusting for traditional cardiovascular risk factors [6,7,8]. Interestingly, cancer was identified as one of the most frequent non-cardiovascular causes of death among participants in several landmark AF trials [4,9]. This emphasizes the dual burden borne by patients with overlapping oncologic and cardiovascular disease, a clinical scenario that is becoming increasingly prevalent as populations age and multimorbidity escalates [9]. POAF is defined, according to the Society of Thoracic Surgeons (STS) and European Society of Cardiology (ESC) guidelines, as new-onset atrial fibrillation or atrial flutter occurring after cardiac surgery in patients with no prior history of arrhythmia, typically within the first 4 weeks of the operation [10,11]. Most cases arise within the first 2–4 days after surgery, coinciding with peak perioperative stress and inflammation.

POAF after cardiac surgery in cancer patients is particularly informative. AF occurring soon after surgery can signal systemic stress responses or underlying cardiac dysfunction [12,13,14]. The inflammatory cascades in cancer—such as cytokine release, NLRP3 inflammasome activation, and epicardial adipose tissue expansion—further promote atrial remodeling and arrhythmogenic substrate formation [15,16].

Clinical management of AF in cancer is challenging. Identifying patients who may benefit from surveillance is debated, especially in cancer populations with limited life expectancy and complex comorbidities [17]. Treatment decisions must balance thromboembolic protection with bleeding risk, especially during periods of cytopenia or invasive therapy, and consider drug–drug interactions [18].

This review aims to (A) emphasize the association between cancer and POAF, (B) explore the underlying pathophysiological mechanisms linking malignancy and arrhythmogenesis, (C) evaluate prognostic implications in cancer patients undergoing cardiac surgery, and (D) propose multidisciplinary strategies for effective screening, prophylaxis, and postoperative management.

## 2. Epidemiology

The intersection of **POAF** and malignancy is increasingly recognized as a clinically relevant and prognostically important association, yet robust epidemiological data remain relatively limited, especially outside the postoperative context.

**Figure 1:**
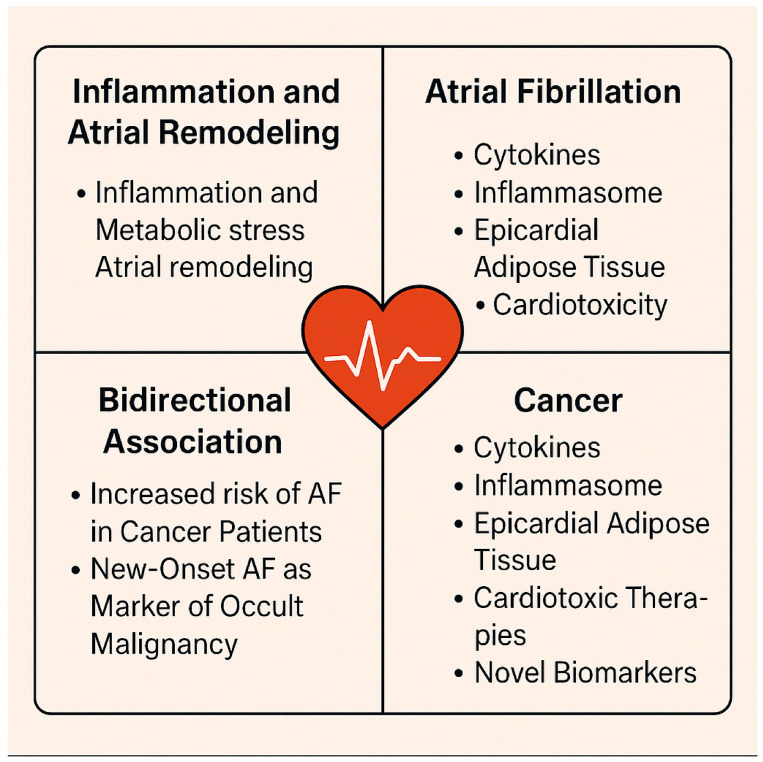
Mechanistic Links Between Atrial Fibrillation and Cancer. This schematic illustrates the interplay between cancer-related processes and atrial fibrillation (AF) development. The top-left quadrant highlights the role of inflammation and metabolic stress in promoting atrial remodeling. The top-right focuses on direct AF mechanisms, including cytokine signaling, inflammasome activation, and epicardial adipose tissue expansion. The bottom-right outlines cancer-specific contributors such as cardiotoxic therapies and emerging biomarkers (e.g., galectin-3, IL-6). The bottom-left emphasizes the bidirectional association: cancer increases AF risk, and NOAF may act as a paraneoplastic indicator. The central heart icon symbolizes the shared cardiovascular-inflammatory axis between AF and malignancy.

Meta-analysis data reveal that cancer survivors have approximately a 47% higher risk of developing AF compared to non-cancer controls [19,20,21]. For instance, Yun et al. analyzed over 800,000 cancer patients in South Korea and reported a 1.63-fold increased risk of AF (adjusted subdistribution hazard ratio, aHR: 1.63), with the strongest associations seen in multiple myeloma (aHR 3.34) and esophageal cancer (aHR 2.69) [19,22]. Comparable findings from a Danish registry support elevated AF risk in hematologic and thoracic cancers, reinforcing that the heterogeneity of cancer-specific risk is likely related to tumor biology, inflammation, and treatment exposures (Figure 1) [23,24]. AF incidence is highest in the immediate 90 days post-cancer diagnosis—possibly reflecting surveillance bias, peri-diagnostic stress, or treatment initiation—while POAF after thoracic oncology surgery likely stems from direct cardiac irritation, autonomic shifts, and metabolic stress [18,24].

A pivotal cohort study by Erichsen et al. involving 24,125 newly diagnosed cancer patients revealed that 2.4% had pre-existing AF at the time of diagnosis, while 1.8% developed NOAF during follow-up, highlighting a significant temporal correlation between cancer diagnosis and arrhythmia onset Critically, the development of NOAF was independently associated with a 1.98-fold increased risk of thromboembolic events and a 6.3-fold increase in incident heart failure, even after adjusting for traditional cardiovascular risk factors (*p* < 0.001) [4,6,25]. These findings underscore the prognostic implications of AF in cancer patients, suggesting that rhythm disturbances may not merely be incidental but serve as markers of cardiovascular stress or systemic disease progression.

Further epidemiological support comes from the Taiwan National Health Insurance Research Database, which examined over 800,000 cancer patients and demonstrated a 47% higher risk of developing AF compared to age- and sex-matched non-cancer controls (adjusted hazard ratio [HR]: 1.47) [26]. Notably, the AF risk varied by cancer type, with hematologic, thoracic, and gastrointestinal malignancies showing the strongest associations [26]. Similar results were reported in a Danish registry-based study involving over 300,000 cancer patients, where hematologic cancers and lung cancers were most significantly associated with AF onset [19,27].

Finally, certain systemic cancer therapies are especially arrhythmogenic. Anthracyclines, alkylating agents, HER2-targeted regimens, and BTK inhibitors like ibrutinib markedly elevate AF risk. Ibrutinib, for example, carries up to a tenfold increased risk of AF, commonly emerging within three months of therapy start [28,29]. Importantly, most of the large-scale studies emphasize a temporal association between cancer diagnosis and AF onset, particularly within the first 90 days of diagnosis, suggesting a multifactorial interplay involving systemic inflammation, surgical stress, metabolic derangement, and direct cardiotoxicity of oncologic treatments [6,26]. This “early AF window” has clinical significance, as it may offer a time-sensitive opportunity for preventive strategies, rhythm monitoring, and risk stratification in newly diagnosed cancer patients.

Conversely, several smaller studies have not corroborated a significant link between cancer and AF. In one such cohort of 131 patients, malignancy was not identified as an independent risk factor for atrial arrhythmias [1]. Likewise, in a prospective analysis of 175 patients with colorectal cancer, AF was not independently associated with survival outcomes [6,30]. These conflicting findings may be attributed to heterogeneity in methodology, limited sample sizes, variation in cancer staging, and differences in AF detection protocols—ranging from intermittent electrocardiography to symptom-based diagnosis. The latter can lead to underestimation of asymptomatic or paroxysmal AF, particularly in patients receiving cardiotoxic therapies that promote subclinical arrhythmic events (Figure 1).

Overall, there is compelling epidemiological evidence supporting the association between AF and cancer. Future investigations, however, should also explore how cancer-specific variables such as tumor type and burden, systemic inflammation, cachexia, and treatment-related cardiotoxicity influence arrhythmia development and long-term cardiovascular outcomes.

## 3. Pathophysiology of AF

Despite significant progress in management strategies, the underlying pathophysiological mechanisms of atrial fibrillation (AF) remain incompletely understood. As a result, many pharmacological and procedural treatments are still based on empirical evidence rather than mechanistic precision [31].

### 3.1. Atrial Myopathy as an AF Substrate

There is growing recognition that AF may represent both a cause and a consequence of an underlying progressive atrial cardiomyopathy [32,33]. This may help explain instances where thromboembolic events are not temporally linked to documented AF episodes in patients with implanted cardiac monitors. As the disease progresses from paroxysmal to persistent AF, the role of Pulmonary Vein (PV) triggers may diminish while atrial fibrosis and non-PV mechanisms become more prominent. Clinical observations indicate significant variability in disease courses: patients experience AF because of atrial fibrosis, while others develop structural changes only after long-standing arrhythmia. Rapid progression of the fibrotic substrate is more frequently observed in women [31]. Obesity, obstructive sleep apnea, hypertension, alcohol intake, and yet-to-be-identified factors may also contribute to this remodeling [33].

Fibrosis is strongly associated with increased AF susceptibility [32]. Ironically, catheter ablation intentionally creates atrial scar tissue to electrically isolate arrhythmic foci. If AF arises predominantly from PV triggers, early ablation may mitigate structural deterioration. However, in patients with diffuse atrial fibrosis, ablation may be less effective. Homogenization of fibrotic regions is being investigated as a therapeutic strategy. Additionally, subepicardial adipose tissue has been shown to undergo fibrotic transformation—potentially driven by immune responses—which may further promote AF [32].

### 3.2. Role of Inflammation and Oxidative Stress

AF is closely associated with inflammatory responses and oxidative stress, particularly in postoperative settings. Elevated levels of inflammatory biomarkers such as C-reactive protein (CRP), tumor necrosis factor-alpha (TNF-α), and interleukins (IL-2, IL-6, IL-8) have been observed in patients with AF [34]. Inflammatory processes may also promote thrombogenesis through endothelial injury, increased tissue factor expression, and platelet activation. Chronic low-grade inflammation, especially in obesity, may induce atrial remodeling. Emerging evidence links viral infections—including HIV—with AF pathogenesis [35].

### 3.3. Impaired Proteostasis in AF

Disruptions in protein homeostasis, such as amyloid deposition composed of atrial natriuretic peptide, have been documented in elderly patients and linked to persistent AF [36]. Heat shock proteins (HSPs), which function to stabilize protein structure and prevent aggregation, are downregulated as AF progresses. Their protective role suggests that restoring HSP function may offer therapeutic benefits, although this remains under investigation [37].

### 3.4. Neurocardiac Remodeling and Autonomic Dysfunction

The autonomic nervous system plays a key role in AF onset and maintenance. Episodes may be triggered by heightened sympathetic or parasympathetic activity. Ganglionated plexi, especially those near the PV ostia, have been explored as ablation targets, although outcomes remain controversial. Atrial pacing and structural heart disease can lead to abnormal nerve sprouting, which is more prominent in the left atrium and may contribute to AF perpetuation [38].

### 3.5. Genetic Factors

A familial predisposition to AF has been confirmed, with multiple ion channelopathies implicated in monogenic forms of the disease [34,39]. Genome-wide association studies (GWAS) have identified over 100 loci linked to AF risk [40,41]. One of the most significant regions lies on chromosome 4q25, near the PITX2 gene, which is involved in pulmonary vein development and left atrial identity [42,43,44]. Further research is focusing on the biological pathways mediated by these variants and their potential utility in precision medicine approaches.

### 3.6. Cancer and Specific Factors

**Figure 2:**
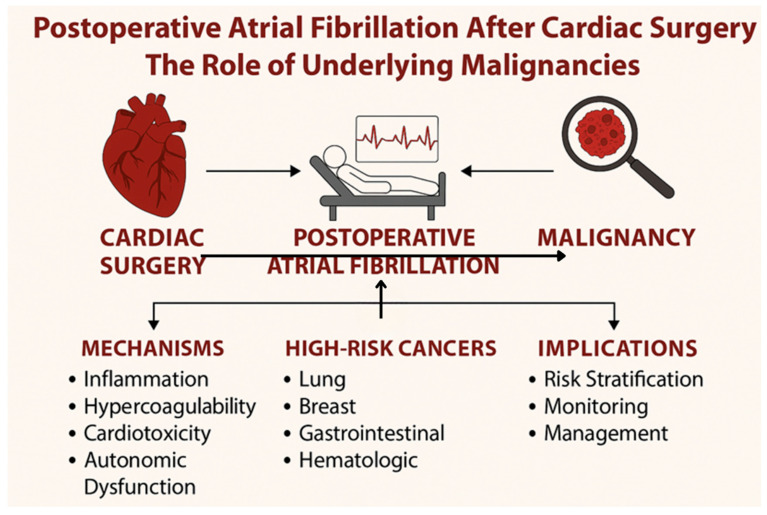
Schematic representation of the relationship between cardiac surgery, underlying malignancy, and the development of POAF. Malignancies may predispose to POAF via systemic inflammation, hypercoagulability, cardiotoxic effects of cancer therapies, and autonomic dysfunction. High-risk cancer types include lung, breast, gastrointestinal, and hematologic malignancies. This association has important clinical implications for risk stratification, postoperative monitoring, and individualized management strategies in patients undergoing cardiac surgery.

Cancer-specific therapies such as anthracyclines, HER2 inhibitors, androgen deprivation, and immune checkpoint inhibitors impose an additional cardiovascular burden (Figure 2) [45,46]. These treatments increase myocardial stress and disrupt cardiac electrophysiology, compounding the proinflammatory and thrombotic environment already present in malignancy [47,48,49].

Furthermore, cancer patients frequently suffer from anemia, malnutrition, or cachexia, which can enhance their vulnerability to arrhythmia. Elevated inflammatory biomarkers such as C-reactive protein (CRP) and interleukin-6 (IL-6) are common in this population and may serve as predictors for POAF [50,51]. Additionally, cancer-associated hypercoagulability likely contributes to a higher risk of embolic events, including ischemic stroke [52].

The interaction between aging, cancer, and cardiovascular disease further intensifies the risk of POAF (Table 1. With aging populations and rising multimorbidity, the coexistence of cancer and cardiovascular disease requires integrated, multidisciplinary approaches [52].

## 4. Clinical Implications and Risk Stratification

Accurate prediction of atrial fibrillation (AF) is essential for identifying at-risk individuals and facilitating early intervention or preventive strategies. Several validated clinical tools, cohort-derived models, and emerging artificial intelligence (AI) systems have been developed to stratify AF risk with increasing precision. However, current risk stratification tools, such as CHA_2_DS_2_-VASc [53], do not incorporate oncologic history or cancer treatments, pointing to the need for cancer-specific scoring systems [5,54,55].

Advanced imaging, such as left atrial strain analysis with speckle-tracking echocardiography, offers predictive insights into POAF risk and should be integrated into perioperative cardiac assessments. Emerging biomarkers—galectin-3 and interleukin-6 (IL-6)—linked to cancer-related inflammation also appear to modulate atrial remodeling and arrhythmia susceptibility [56,57].

Additionally, cardio-oncology has spotlighted arrhythmogenic risks of cancer treatments—immune checkpoint inhibitors and tyrosine kinase inhibitors such as ibrutinib—both increasingly implicated in AF development [58,59].

Multidisciplinary collaboration—involving cardiologists, oncologists, anesthesiologists, and surgeons—is essential to optimize perioperative care. In some patients, prophylactic antiarrhythmic strategies should be considered based on both cardiovascular and oncologic risk factors. It is encouraging that in recent years, we have seen the advent of machine learning (ML) and artificial intelligence (AI) models capable of outperforming traditional risk scores in specific contexts. ML-based systems, including gradient boosting algorithms, have demonstrated superior performance to CHA_2_DS_2_-VASc in stroke prediction and bleeding risk stratification [60,61]. Deep learning applied to ECG data can identify subclinical patterns predictive of future AF with an area under the curve (AUC) ranging from 0.75 to 0.8 [62].

### Temporal and Prognostic Interplay Between AF and Malignancy

Recent evidence suggests that the relationship between AF and cancer may be bidirectional. New-onset atrial fibrillation (NOAF) has been shown to occasionally precede the diagnosis of malignancy, raising the possibility that it may serve as a paraneoplastic manifestation or an early clinical harbinger of occult cancer [6,27]. A large Danish registry study found a significantly elevated risk of cancer diagnosis within three months of incident AF, indicating a potential pathophysiological or diagnostic overlap [6].

## 5. Management in Cancer Patients

Oncologic history, including cancer type, stage, and prior therapies, must be considered alongside conventional cardiac risk factors during preoperative evaluation. Standard risk tools such as CHA_2_DS_2_-VASc fail to account for the influence of cancer or its treatments and may underestimate true arrhythmic risk in these patients [53].

Cancer patients may benefit from intensified monitoring strategies such as continuous telemetry for at least the first 5–7 days postoperatively. Close surveillance for arrhythmia onset is particularly warranted in high-risk patients with hematologic, lung, gastrointestinal, or breast malignancies [1,6].

### Medical Treatment

Emerging data suggests that inflammation-modulating agents like colchicine or corticosteroids may reduce POAF incidence, especially in patients with elevated inflammatory markers (e.g., CRP, IL-6) [4,63,64]. Beta-blockers, amiodarone, and statins may also be considered on a case-by-case basis for prophylaxis.

An integrated care model involving cardiologists, oncologists, anesthesiologists, and cardiac surgeons is essential. Decisions about surgical timing, oncologic treatment interruption, and rhythm management should be individualized based on cancer activity, cardiovascular status, and overall prognosis [63,64,65,66].

The medical treatment of established POAF primarily focuses on ventricular rate control and hemodynamic stabilization. Beta-blockers are the first-line therapy, as extensive evidence—including randomized trials and meta-analyses—demonstrates a significant reduction in POAF incidence by approximately 60–70% following cardiac surgery, (RR 0.43–0.61) [67,68]. Both the ESC and AHA/ACC guidelines recommend perioperative beta-blocker use as a Class I intervention for POAF prevention [69,70]. In patients with contraindications to beta-blockers (e.g., bradycardia or low output states), alternatives such as diltiazem or verapamil may be used, while electrical cardioversion is indicated in cases of hemodynamic instability.

When rhythm control is indicated, amiodarone is frequently employed either as rescue therapy or as part of a long-term antiarrhythmic regimen. Randomized data suggests that combination therapy with amiodarone and beta-blockers reduces the incidence and delays the onset of POAF (e.g., 5.95% vs. 9.25%, HR 0.27, *p* = 0.006) [71]. The ESC 2024 guidelines endorse this combination as a Class I recommendation in high-risk patients [72]. Nevertheless, long-term use of amiodarone may result in adverse effects such as pulmonary fibrosis or thyroid dysfunction. In patients without structural heart disease, propafenone or flecainide may be considered with caution. Anticoagulation decisions should follow CHA_2_DS_2_-VASc scoring and are especially critical when POAF episodes last longer than 48 h or when cardioversion is planned [10].

Pulmonary vein isolation (PVI) remains the cornerstone of catheter ablation for AF and has been applied in the setting of refractory POAF, although with nuanced results. The pathophysiology of POAF often involves both PV–related triggers and non-pulmonary sources of atrial ectopy, especially in the context of postoperative inflammation and atrial injury. While PVI demonstrates high efficacy in paroxysmal AF (80–90% success), its long-term effectiveness in post-cardiac surgery patients—many of whom exhibit atrial fibrosis or conduction abnormalities—is reduced. Late recurrences following PVI are common and are typically attributed to pulmonary vein reconnection, progressive atrial remodeling, or activation of non-PV triggers such as the posterior wall or left atrial appendage. Studies exploring adjunctive strategies—including ablation of the ligament of Marshall, complex fractionated electrograms, or low-voltage areas—have yielded mixed results and are not yet standard of care. Therefore, while PVI represents a foundational technique in the management of POAF, further research is needed to optimize lesion sets, enhance procedural durability and determine patient subsets most likely to benefit from this approach [62,73,74].

These models offer promise for early AF detection in asymptomatic individuals. It is increasingly evident that malignancy serves as a significant, albeit underrecognized, risk factor for the development of NOAF in the postoperative setting, particularly following cardiac surgery. This association is biologically plausible given that both cancer and AF are linked through shared pathophysiological pathways, including systemic inflammation, oxidative stress, autonomic dysfunction, metabolic dysregulation, and endothelial injury [6,27,75].

Moreover, the cardiotoxic effects of contemporary oncologic therapies—including anthracyclines, HER2-targeted agents, immune checkpoint inhibitors, and thoracic radiotherapy—can directly impair myocardial structure and function, thereby predisposing to electrical instability and AF [75,76,77,78]. Thoracic irradiation and chemotherapeutic regimens are known to induce myocardial fibrosis, microvascular ischemia, and autonomic imbalance, all of which amplify the susceptibility to arrhythmogenesis [27,76,78].

## 6. AF Triggers, Substrates, and Ablation Strategies

PVI remains the cornerstone of AF ablation therapy, following the pivotal work [73], which demonstrated that ectopic foci within the PVs trigger AF [73]. PVI is particularly effective in patients with paroxysmal AF, achieving success in approximately 80–90% of cases. However, this efficacy decreases significantly in patients with persistent AF or structural heart disease. The utility of additional ablation beyond PVI—such as targeting the ligament of Marshall, superior vena cava, left atrial appendage (LAA), complex fractionated electrograms, or fibrotic zones—remains an area of active investigation. Rotor mapping and ablation have shown inconsistent clinical results across studies [79]. Techniques currently range from segmental PVI to more extensive substrate modification, yet their long-term effectiveness remains variable and unresolved.

## 7. Durability and Recurrence of AF After PVI

Despite initial success, late recurrence of AF following PVI is common and may occur due to PV reconnection, presence of non-PV triggers, or progressive remodeling and fibrosis of the atrial substrate. Improving the long-term durability of lesion sets and understanding mechanisms of recurrence remain critical goals in AF research [69].

## 8. Conclusions, Future Directions and Research Gap

This review identifies malignant **POAF** following cardiac surgery [29]. The integration of oncologic variables into cardiovascular care remains underemphasized in current guidelines, despite mounting evidence that cancers—particularly lung, breast, gastrointestinal, hematologic, and prostate—contribute to arrhythmogenesis via systemic inflammation, cardiotoxicity, autonomic dysfunction, and metabolic disturbances [48,51,52,55,79,80,81].

*Multidisciplinary collaboration*—involving cardiologists, oncologists, anesthesiologists, and surgeons—is essential to optimize perioperative care. In some patients, prophylactic.

Antiarrhythmic strategies should be considered based on both cardiovascular and oncologic risk factors.

Despite emerging data, limitations remain. Much of the current evidence comes from observational studies that lack cancer-specific stratification or do not account for the timing of cancer diagnosis, therapy, and POAF development. To improve clinical decision-making, *well-designed prospective trials* are needed to validate prevention and management strategies tailored for cancer patients undergoing cardiac surgery.

Cancer significantly contributes to the risk of POAF following cardiac surgery, driven by a complex interplay of inflammatory, thrombotic, metabolic, and treatment-related factors. 

Among various malignancies, lung, breast, gastrointestinal, hematologic, and prostate cancers exhibit the strongest associations with POAF, primarily due to their systemic inflammatory burden and the cardiotoxic potential of standard oncologic therapies (Table 1) [19,45,47,48].

Recognizing cancer as a non-traditional yet pivotal risk factor for POAF calls for a paradigm shift in perioperative risk assessment. Traditional evaluation strategies should expand to include comprehensive oncologic reviews—accounting for prior and ongoing therapies that influence arrhythmic vulnerability [49].

Clinically, the implications are substantial. Tailored approaches such as prophylactic antiarrhythmic therapy, enhanced monitoring of inflammatory biomarkers, and structured collaboration between oncology and cardiology teams could significantly reduce POAF incidence and enhance recovery. Early identification of at-risk cancer patients may inform surgical timing, guide therapy modifications, and prompt intensified postoperative rhythm surveillance [5,49,63].

Despite growing awareness, critical knowledge gaps remain. The heterogeneity in cancer types, treatment exposures, and patient comorbidities complicates the creation of universal risk models. Thus, there is a pressing need for predictive tools that incorporate oncologic variables, research into novel cardiac stress biomarkers specific to malignancy, and prospective trials targeting prevention and management of POAF in cancer patients [49,82].

Ultimately, understanding the intersection of oncology and cardiac surgery represents a major step toward precision medicine. Integrating patient-specific cancer-related data into perioperative.

Planning is essential to minimizing complications and optimizing long-term cardiovascular and oncologic outcomes.

*A critical gap* in the current literature is the scarcity of stratified data differentiating cancer types (solid versus hematologic) and disease activity (active versus history) in POAF cohorts. However, broader epidemiologic studies on AF offer valuable insights that can inform this discussion:

- Hematologic malignancies, including multiple myeloma (adjusted subdistribution hazard ratio [aHR]: 3.34), leukemia (aHR: 2.64), and lymphoma (aHR: 2.29), have been consistently associated with markedly increased AF risk compared with non-cancer controls [53].

Among solid tumors, intrathoracic cancers—such as lung, esophageal, and central nervous system (CNS) malignancies—demonstrate similarly elevated risks (e.g., esophageal cancer: aHR ≈ 2.69; CNS cancer: ≈ 2.62; lung cancer: ≈ 2.39 [26].

A population-based cohort from Taiwan found that solid cancers such as esophageal, lung, and gallbladder cancers exhibited the highest AF incidence rates—most notably in older males—whereas thyroid cancers showed much lower rates [82].

These data suggest that the strength of association between cancer and POAF likely varies by cancer subtype, with hematologic and intrathoracic solid tumors potentially driving much of the observed risk. Nonetheless, data stratifying by active versus historical cancer status remain scarce, particularly in perioperative settings such as POAF following cardiac surgery.

Future studies should address this gap by collecting detailed data on cancer subtypes (solid vs. hematologic), location (e.g., intrathoracic), disease status (active vs. in remission), and treatment exposures. Such stratification would clarify which subgroups are most vulnerable to POAF, enhance understanding of underlying pathophysiological mechanisms (e.g., inflammation, cardiotoxicity), and support tailored perioperative management strategies.

Despite growing awareness, the current literature on POAF in cancer patients undergoing cardiac surgery remains limited by retrospective cohorts, small sample sizes, inconsistent definitions, and lack of oncologic stratification. Research priorities that can advance precision cardio-oncology include.

1. Integrated Predictive Models
  ○ Develop and validate multivariable risk models that incorporate oncologic parameters (tumor type, stage, treatment history, systemic inflammation, prothrombotic state) alongside traditional cardiac variables such as age, left atrial enlargement, heart failure, and hypertension [83].
  ○ Emulate efforts like the “POAF Score + biomarker panel” approach, which significantly improved prediction over clinical factors alone [84].
2. Prospective Cohorts & Registries
  ○ Launch multicenter, prospective studies with standardized definitions and detailed phenotyping (cancer type, timeline, treatment exposures).
  ○ Build dedicated cardio-oncology surgical registries to generate real-world evidence and support TRIPOD-compliant model development [85].
3. Biomarker Discovery & Validation
  ○ Investigate serum and pericardial biomarkers—such as BNP, troponins, microRNAs, extracellular vesicles, circulating tumor DNA (ctDNA), systemic immune-inflammation index (SII), and others—for early identification of cardiac stress and arrhythmogenic remodeling.
4. Mechanistic Translational Research
  ○ Conduct preclinical, cellular, and electrophysiologic studies to explore cancer-mediated atrial vulnerabilities, including inflammation, fibrosis, connexin dysregulation, oxidative stress, and autonomic imbalance.
5. Interventional Trials in High-Risk Patients
  ○ Test targeted cardioprotective strategies (e.g., beta-blockers, amiodarone, colchicine, steroids, ARNI therapy) in randomized settings among cancer patients at elevated risk [85].
  ○ Validate innovative approaches like sacubitril/valsartan, which showed promise in non-oncologic cohorts [86].
6. Tailored Perioperative Protocols
  ○ Create perioperative care algorithms specific to cancer patients, incorporating optimal surgical timing around chemotherapy, fluid/electrolyte balance, drainage strategies, and thromboprophylaxis.
  ○ Employ multidisciplinary preoperative cardio-oncology evaluations to individualize management.
7. Long-Term Outcomes & Survivorship Monitoring
  ○ Study the long-term impact of POAF on recurrence-free survival, cardiovascular and thromboembolic events, and overall survival in cancer survivors. NOAF is associated with >3-fold increased risk of chronic AF [79].
8. Digital Health & ML-Driven Risk Tools
  ○ Incorporate wearable monitoring, ECG-based deep learning, and administrative data into real-time perioperative risk mitigation systems [80].

Another critical dimension to consider is the role of cardiac surgical procedure type in influencing POAF. Across multiple cohorts, the incidence of POAF varies significantly by operation: approximately 20–30% following isolated Coronary Artery Bypass Grafting (CABG), rising to 35–40% after isolated valve surgery, and climbing to 38–63%—or even up to 50% after combining CABG and valve procedures [81]. Furthermore, one large registry reported a POAF incidence of 25.2% in CABG patients versus 49.0% in those undergoing valve surgery [87].

In the context of cancer-associated POAF, these procedure-specific nuances are rarely addressed. It is plausible that the confluence of surgical complexity (e.g., prolonged cardiopulmonary bypass or atriotomy) and cancer-related pro-inflammatory or prothrombotic states may compound arrhythmia risk, yet studies seldom stratify outcomes by both cancer status and surgical type. Acknowledging this gap is important for clinical applicability.

We recommend that future research incorporate procedure-specific analyses (such as CABG, valve-only, and combined surgeries) when examining cancer-associated POAF. This stratification could reveal subgroup-specific vulnerabilities and support tailored preventive strategies and perioperative management.

POAF remains one of the most frequent complications after cardiac surgery and is increasingly recognized as being influenced by underlying malignancy. While the mechanisms linking cancer and POAF are multifactorial—including inflammation, metabolic alterations, treatment-related cardiotoxicity, and surgical stress—the clinical implications are substantial.

Based on current evidence, we suggest several practical strategies for clinicians:

1. Closer postoperative surveillance of patients with a history of cancer or active malignancy, particularly following high-risk procedures such as valve or combined surgeries.
2. Consideration of targeted cancer screening in patients with new-onset POAF after cardiac surgery, especially if other risk factors are absent, as arrhythmia may occasionally be a marker of occult malignancy.
3. Integration of POAF into oncologic risk assessment—clinicians should recognize POAF as a potential “red flag” event that warrants multidisciplinary discussion, including cardiology and oncology input.
4. Individualized perioperative management, including optimization of inflammation and fluid balance, and careful adjustment of antiarrhythmic or anticoagulation strategies in patients with concurrent cancer.

Future studies should refine risk stratification by cancer type, disease stage, and surgical procedure to guide precision strategies in this complex population. Until such data become available, heightened clinical vigilance, early recognition, and cross-specialty collaboration remain the most practical approaches to improving outcomes in patients with cancer-associated POAF.

## Figures and Tables

**Table 1:** Association Between Cancer Types and POAF Risk.

Cancer Type	Mechanisms	Notable Treatment Affecting Risk	Prevalence/Impact
Lung Cancer	Chronic inflammation, Hypercoagulability	Chemotherapy, Radiotherapy	Highest risk among all cancers
Breast Cancer	Cardiotoxicity from treatment	Anthracyclines, HER2-targeted therapies	Increased risk, especially with therapy
Gastrointestinal Cancers	Inflammation, Metabolic disturbances	Various chemotherapy regimens	Moderate; associated with inflammation
Hematologic Malignancies	Systemic inflammation, Cytokine release	Chemotherapy, Immunotherapy	Variable; cytokine-mediated AF triggers
Prostate Cancer	Cardiovascular complications from ADT	Androgen deprivation therapy (ADT)	Low to moderate risk; ADT-related

## Data Availability

Data are contained within this article.
